# Exploring the Wound‐Healing Properties of *Anchusa azurea*: A Nanoliposome‐Based Approach

**DOI:** 10.1002/open.70254

**Published:** 2026-08-13

**Authors:** Sevim Feyza Erdoğmuş, Hasan Hüseyin Demirel, Özlem Erdal Altıntaş, İffet İrem Tatlı Çankaya, Nurullah Okumuş

**Affiliations:** ^1^ Department of Basic Pharmaceutical Sciences Faculty of Pharmacy Afyonkarahisar Health Sciences University Afyonkarahisar Turkey; ^2^ Bayat Vocational School Department of Laboratory and Veterinary Health Afyon Kocatepe University Afyonkarahisar Turkey; ^3^ Department of Nutrition and Dietetics Faculty of Health Sciences Afyonkarahisar Health Sciences University Afyonkarahisar Turkey; ^4^ Department of Pharmaceutical Botany Faculty of Pharmacy Hacettepe University Ankara Turkey; ^5^ Faculty of Medicine Department of Pediatrics Afyonkarahisar Health Sciences University Afyonkarahisar Turkey

**Keywords:** *Anchusa azurea*, antioxidant activity, nanoliposome, phenolic compounds, wound healing

## Abstract

*Anchusa azurea* has attracted increasing interest as a medicinal plant due to its phenolic and flavonoid compounds, which may contribute to its antioxidant and wound‐healing activities. In this study, aqueous (AW) and aqueous ethanolic (AE) extracts obtained from the aerial parts of *Anchusa azurea* by ultrasonic extraction were evaluated for their phytochemical composition, antioxidant activity, cytotoxicity, and wound‐healing efficacy using in vitro and in vivo models. The AE extract exhibited higher total phenolic and flavonoid contents and stronger antioxidant activity than the AW extract. Cytotoxicity was assessed on human skin fibroblasts, adult (HDFa) cells, using the MTT assay. Both extracts showed no significant cytotoxic effects. AW‐loaded nanoliposomes (AWNP) and AE‐loaded nanoliposomes (AENP) were successfully prepared, and scanning electron microscopy, transmission electron microscopy, and dynamic light scattering analyses confirmed nanoscale vesicles with negative zeta potential values. In vitro release studies demonstrated biphasic release profiles, with AENP exhibiting greater cumulative release than AWNP. In a rat excisional wound model, nanoliposomal formulations significantly enhanced wound contraction compared with crude extracts and controls. Histopathological evaluation demonstrated increased collagen deposition, neovascularization, and re‐epithelialization, particularly in the AE and AENP groups. These findings suggest that the phenolic‐rich aqueous ethanolic extract and its nanoliposomal formulation are promising candidates for wound‐healing applications.

## Graphical abstract

*Anchusa azurea* extracts were prepared by ultrasonic extraction method. Phytochemical composition, antioxidant activity, cytotoxicity, and wound‐healing efficacy of the plant extracts using *in vitro* and *in vivo* models. The plant extracts loaded nanoliposomes were successfully obtained and characterized by scanning electron microscopy, transmission electron microscopy, dynamic light scattering, and zeta potential analyses. The *in vivo* wound healing effects of the extracts and their nanoliposomal formulations were evaluated.

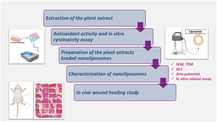

## 1 Introduction

Wound healing is a complex and highly dynamic biological process involving a tightly regulated sequence of molecular and cellular events that ultimately result in tissue regeneration. This process requires precise spatial and temporal coordination among multiple cell types, each contributing to the overlapping phases of inflammation, proliferation, including neoangiogenesis, granulation tissue formation, re‐epithelialization, and maturation through extracellular matrix (ECM) remodeling [1, 2, 3, 4]. Disruption of any of these phases may lead to delayed healing or chronic wounds, making wound management a major therapeutic and economic challenge within modern healthcare systems [5]. Despite the continuous development of advanced wound care products, there is still no consensus regarding the essential characteristics of an ideal wound dressing capable of fully supporting tissue regeneration under diverse pathological conditions [6, 7, 8].

Medicinal plants have long been recognized as valuable resources in wound management due to their ability to modulate one or more stages of the healing process, maintain a moist wound environment, and provide protection against microbial infection [9]. Their antimicrobial, anti‐inflammatory, and antioxidant properties collectively contribute to the acceleration of wound repair. Among these mechanisms, antioxidant activity plays a particularly critical role by neutralizing excessive reactive oxygen species (ROS) generated during prolonged or chronic inflammation. Uncontrolled ROS production following the phagocytosis of necrotic tissue by neutrophils and macrophages during the inflammatory phase can impair cellular migration, collagen synthesis, and re‐epithelialization, ultimately delaying the healing process [10].

*Anchusa azurea* Mill., a perennial species belonging to the Boraginaceae family, has long been utilized in Turkish folk medicine, particularly for the treatment of wounds, various skin disorders, and as a diuretic agent. Ethnopharmacological records indicate that several *Anchusa* species are traditionally applied topically to promote wound closure and tissue regeneration, highlighting their potential therapeutic relevance and the need for scientific validation. Phytochemical investigations of *A. azurea* have revealed a rich composition of bioactive secondary metabolites, including phenolic acids, flavonoids, and naphthoquinone derivatives, which are widely recognized for their potent antioxidant and anti‐inflammatory activities [11]. Furthermore, quantitative analyses have demonstrated that *A. azurea* contains considerable levels of endogenous antioxidants, such as reduced and oxidized glutathione (GSH and GSSG) as well as vitamin C, all of which play critical roles in protecting tissues against oxidative stress during the wound‐healing process [12].

Oxidative stress and prolonged inflammation are among the principal factors that impair normal wound repair; therefore, the pronounced antioxidant and anti‐inflammatory capacity of *A. azurea* provides a plausible mechanistic basis for its traditional medicinal use. Previous pharmacological studies have reported that extracts of *A. azurea* and its major phenolic constituent, rosmarinic acid, exhibit significant anti‐inflammatory activity in experimental models, primarily through the modulation of pro‐inflammatory mediators [13]. More recently, in vivo evidence supporting the wound‐healing potential of *A. azurea* has begun to emerge. A controlled experimental study employing a rat burn wound model demonstrated that topical application of *A. azurea* methanolic extract significantly enhanced wound contraction, collagen deposition, and re‐epithelialization, while concurrently reducing oxidative damage and inflammatory cytokine levels [14]. However, despite these encouraging findings, studies integrating advanced delivery strategies with comprehensive wound‐healing evaluation remain limited. Although previous studies have reported the presence of antioxidant constituents in *A. azurea*, comprehensive characterization of its antioxidant capacity and bioactive composition in relation to wound‐healing activity remains limited. Since phenolic and flavonoid compounds are known to contribute significantly to antioxidant defense mechanisms and tissue repair, the determination of total phenolic and flavonoid contents together with multiple antioxidant assays may provide valuable insight into the biological basis of the wound‐healing potential of *A. azurea* extracts.

In recent years, drug delivery systems have attracted increasing attention due to the challenges associated with delivering bioactive compounds effectively to target tissues [15, 16, 17, 18]. Conventional formulations may fail to achieve adequate local concentrations or may result in undesirable systemic exposure. In contrast, controlled drug delivery systems offer several advantages, including sustained release, improved stability of active compounds, reduced dosing frequency, and prolonged retention at the site of application. Among these systems, nanoparticles are widely utilized due to their structural and functional versatility, allowing active substances to be dissolved, encapsulated, adsorbed, or conjugated to their surfaces [19, 20]. Their nanoscale size facilitates enhanced tissue penetration, while biodegradable materials enable controlled and sustained release profiles. Within this context, nanoliposome‐based delivery systems are particularly attractive for wound‐healing applications, especially for plant‐derived extracts [21, 22]. Unlike many other nanocarrier systems, nanoliposomes possess a phospholipid bilayer structure that enables the simultaneous encapsulation of both hydrophilic and lipophilic bioactive compounds, making them particularly suitable for complex plant extracts [23]. In addition to improving the physicochemical stability of phytochemicals and protecting sensitive constituents from degradation, nanoliposomes exhibit high biocompatibility, biodegradability, and low toxicity, which are highly desirable characteristics for topical wound‐healing applications [23, 24]. Furthermore, owing to their structural similarity to biological membranes, nanoliposomes can enhance the interaction of bioactive compounds with skin tissues and improve local drug delivery [22]. Previous studies have demonstrated that liposomal formulations can improve the therapeutic efficacy of natural products by increasing their stability, retention at the application site, and bioavailability [25, 26]. These advantages make nanoliposomes a promising delivery platform for maximizing the therapeutic potential of medicinal plant extracts in wound‐healing applications. Nanoliposomes can enhance the penetration of bioactive compounds into damaged tissue, protect sensitive phytochemicals from degradation, and provide localized and controlled release at the wound site, thereby improving therapeutic efficacy and safety [27].

The aim of the present study was to investigate the antioxidant and cytotoxic properties of aqueous and aqueous‐ethanolic extracts of *Anchusa azurea*. Furthermore, the wound‐healing potential of these extracts and their corresponding extract‐loaded herbal nanoliposomes was evaluated using an excisional wound model.

## 2 Materials and Methods

### 2.1 Extraction of the Plant Extract

*Anchusa azurea* Mill. was collected from the Hacettepe University Beytepe Campus (Ankara, Türkiye) in June, and botanical identification was performed by Prof. Dr. İffet İrem Tatlı Çankaya. Prior to extraction, the collected aerial parts of the plant were air‐dried at room temperature in the shade for 2 weeks and protected from direct sunlight to minimize the degradation of thermolabile bioactive compounds. Only the aerial parts of the plant were used for extraction. The dried plant material was ground into a fine powder using a laboratory grinder. AW and AE extracts were prepared by ultrasonic‐assisted extraction as previously described [28]. Briefly, 30 g of powdered plant material was extracted with 400 mL of either distilled water or 50% (v/v) aqueous ethanol at 25°C–37°C for 1 h under ultrasonication. During the extraction process, the temperature was maintained below 40°C to prevent thermal degradation of bioactive constituents. The extracts were filtered through Whatman No. 1 filter paper, and the extraction procedure was repeated 5–6 times. The filtrates obtained from each extraction cycle were combined and concentrated under reduced pressure using a rotary evaporator at temperatures below 40°C. The concentrated extracts were transferred into airtight amber glass bottles, protected from light, and stored at 4°C in a laboratory refrigerator (Arçelik, Türkiye) until further use. The extraction yields were 11.6% and 12.5% for the aqueous (AW) and aqueous ethanolic (AE) extracts, respectively.

### 2.2 Phytochemical Characterization and Antioxidant Activity

The phytochemical characterization and antioxidant properties of AW and AE extracts were evaluated using 2,2‐diphenyl‐1‐picrylhydrazyl (DPPH) radical scavenging, cupric ion reducing antioxidant capacity (CUPRAC), ABTS radical cation scavenging, ferric reducing antioxidant power (FRAP), total phenolic content (TPC), and total flavonoid content (TFC). The assays were carried out in triplicate, and the results were expressed as average values with SD (standard deviation).

#### 2.2.1 DPPH Radical Scavenging Activity

1 mM DPPH radical solution was prepared in ethanol. 50 µL of this solution was mixed with 150 µL of different concentrations of the extract (200 to 12.5 µg/mL) and the reference (200 to 1.25 µg/mL) dissolved in ethanol. The reaction mixture was incubated for 30 min in the dark, then absorbance was measured at 517 nm. Quercetin was used as reference. Radical scavenging activity was expressed as the inhibition percentage and was calculated according to Equation (1) [29].

(1) $$\text{Inhibition }\%=[(A_{\text{blank}}-A_{\text{sample}})/A_{\text{blank}}]\times 100$$

*A*_blank_ is the absorbance of the blank (containing ethanol instead of sample), and *A*_sample_ is the absorbance of the extracts or reference. The half‐maximal inhibitory concentration (IC_50_) value for each extract was calculated from the plotted graph of scavenging activity against the concentrations of the sample.

#### 2.2.2 CUPRAC Reducing Antioxidant Capacity

For the CUPRAC assay, 50 μL CuCl_2_ solution, 50 μL neocuproine solution, and 50 μL ammonium acetate buffer (1 M, pH 7.0) were mixed in a 96‐well microplate. Then 25 µL of extracts or different concentrations of reference (800 to 25 µg/mL) and 25 µL of distilled water were added to the initial mixture, separately. After incubation in the dark for 30 min, absorbance was measured at 450 nm. Gallic acid was used as reference. Results were presented as gallic acid equivalents (mg/g extract) using a calibration curve [29].

#### 2.2.3 ABTS Radical Cation Scavenging Activity

ABTS radical cation solution was prepared by mixing 7 mM ABTS and 2.45 mM potassium persulfate solutions and allowing the mixture to stand in the dark for 12–16 h. The resulting solution was diluted with ethanol to obtain an absorbance of 0.70 at 734 nm. Subsequently, 20 μL of extract solution was mixed with 200 μL of ABTS^+^ solution and incubated for 6 min in the dark. Absorbance was measured at 734 nm. Trolox was used as reference. Results were expressed in terms of Trolox equivalent antioxidant capacity (TEAC) using a calibration curve [29].

#### 2.2.4 FRAP Reducing Antioxidant Capacity

For FRAP analysis, 20 μL of extract solution was mixed with 50 μL of 0.2 M sodium phosphate buffer (pH 6.6) and potassium ferricyanide solution. After incubation at 50°C for 20 min, 50 μL of 10% trichloroacetic acid was added. Subsequently, 50 μL of distilled water and 10 μL of 0.1% ferric chloride solution were added, and the mixture was incubated for 30 min. Absorbance was measured at 700 nm, and quercetin was used as reference. Quercetin was used as reference. Results were presented as quercetin equivalents (mg/g extract) using a calibration curve [29].

#### 2.2.5 Total Phenolic Content

Total phenolic content was determined using the Folin–Ciocalteu method. Briefly, 20 μL of extract solution was mixed with 100 μL of diluted Folin–Ciocalteu reagent and 80 μL of sodium carbonate solution (7.5%). Following incubation in the dark for 2 h, absorbance was measured at 765 nm. Gallic acid was used as reference. The total phenolic contents of the extracts were expressed as gallic acid equivalents (mg/g extract) using a calibration curve [29].

#### 2.2.6 Total flavonoid content

Total flavonoid content was determined using the aluminum chloride colorimetric method. Briefly, 25 μL of extract solution was mixed with ethanol, aluminum chloride, potassium acetate, and distilled water. After incubation for 30 min at room temperature, absorbance was measured at 415 nm. The assay was carried out in triplicate. Quercetin was used as reference. Results were presented as quercetin equivalents (mg/g extract) using a calibration curve [29].

### 2.3 In Vitro Cytotoxicity Assay

The cytotoxicity *of Anchusa azurea* water and aqueous ethanol extracts (AW, AE) on HDFa cell line (ATCC, PCS‐201−012) was determined by MTT assay [30]. MTT assay is a colourimetric method used to determine cell viability. This method is based on the reduction of yellow colored tetrazolium salt to purple colored formazan crystals insoluble in water with the help of dehydrogenase enzymes secreted from mitochondria of metabolically active cells. The density of formazan crystals formed after MTT analysis is proportional to the number of living cells in the medium. Therefore, this method will be used to determine cell viability in in vitro cytotoxicity and cell proliferation studies. Plant extracts were added to the cell culture flasks in twofold increasing concentrations and incubated at 37°C for 24, 48, and 72 h. At the end of incubation, 20 μL MTT solution (5 mg/mL in PBS) was added to each well and incubated at 37°C for 2–4 h. At the end of the time, MTT stain was removed from the cells and 200 µL DMSO was added to each well and incubated for another 5 min. The color change was determined at a wavelength of 570 nm in an ELISA plate reader. Control cell viability was accepted as 100%, and the viability of the experimental cells was determined by proportioning them to the control. The percentage of cell viability was calculated according to Equation (2).

(2) $$\text{Cell viability}\;(\%)=\left[1-\left(\frac{A_{570\;}\text{of the test}}{A_{570\;}\text{of control}}\right)\right]\times 100$$

### 2.4 Preparation of the Plant Extracts Loaded Nanoliposomes and Characterization

Plant extract–loaded nanoliposomes were prepared according to the method described by Khoshraftar et al. [31], with minor modifications. Briefly, 1 g of phosphatidylcholine was dissolved in 30 mL of 96% ethanol under continuous stirring at 50°C for 1 h until complete dissolution of the lipid phase was achieved. The organic solvent was subsequently removed using a rotary evaporator (Heidolph, Germany) to obtain a thin phosphatidylcholine film. Each plant extract (1.5 g) was separately dissolved in 36 mL of phosphate‐buffered saline (PBS) at ambient temperature. The resulting aqueous extract solution was then added to the phosphatidylcholine film and gently mixed to allow hydration of the lipid layer. The obtained suspension was subjected to sonication in an ultrasonic bath for 2 min at 40°C to reduce particle size and promote nanoliposome formation. The prepared nanoliposomes were subsequently dried in an oven dryer at 60°C for 48 h. The physicochemical characterization of the extract‐loaded nanoliposomes was performed using scanning electron microscopy (SEM) and transmission electron microscopy (TEM) to evaluate surface morphology, particle shape, and internal structural features. Particle size distribution, polydispersity index (PDI), and zeta potential values were determined by dynamic light scattering (DLS) analysis, as previously described [31].

### 2.5 In Vitro Release Study of Plant Extract‐Loaded Nanoliposomes

The in vitro release behavior of plant extract‐loaded nanoliposomes was evaluated using phosphate‐buffered saline (PBS, pH 7.4) as the release medium under physiological conditions (37°C ± 1°C). Prior to the release study, calibration curves for the AW and AE extracts were prepared using the corresponding free (non‐encapsulated) extracts dissolved in PBS at different concentrations. Absorbance measurements were performed at 350 nm for AE and 340 nm for AW extracts using a UV–visible spectrophotometer.

The resulting calibration curves were used for quantification of the released extract. Briefly, nanoliposomal formulations were dispersed in 10 mL PBS (pH 7.4) and incubated in a thermostatically controlled shaking water bath. At predetermined time intervals (15, 30, 60, 120, 180, and 300 min), 1.5 mL aliquots were withdrawn and transferred into Eppendorf tubes for analysis. An equal volume of fresh PBS was immediately added after each sampling to maintain a constant release volume throughout the experiment. The collected samples were analyzed spectrophotometrically, and the cumulative amount of released extract was calculated using the corresponding calibration curve. The release profiles were expressed as cumulative release percentage versus time.

### 2.6 In Vivo Wound Healing Study

Male Wistar albino rats, weighing 250–300 g, were used in this study. Animals were housed under standard laboratory conditions (24°C ± 1°C, 12 h light/12 h dark cycle, and controlled ventilation) with free access to standard pellet diet and water ad libitum. All experimental procedures were carried out in accordance with institutional guidelines for the care and use of laboratory animals and were approved by the local ethics committee.

To evaluate the in vivo wound‐healing efficacy of *Anchusa azurea* extracts and extract‐loaded nanoliposomes (AENP, AWNP), an excisional wound model was established in rats. Prior to wound induction, animals were fasted for 12 h and anesthetized via intraperitoneal administration of ketamine (87 mg/kg) and xylazine (13 mg/kg). The dorsal interscapular region was shaved and disinfected, and a full‐thickness circular excisional wound was created using a 1 cm sterile biopsy punch, ensuring that the wound area did not exceed 10% of the total body surface area [32]. Full‐thickness excisional wounds were confirmed by visual inspection of the subcutaneous fascia layer following biopsy punch application. Hemostasis was achieved by applying gentle pressure with sterile gauze for approximately 30 s, and no suturing was performed in order to allow natural wound healing. Following surgery, animals were housed individually to prevent wound interference and were monitored daily for general health status and signs of wound infection. No topical antiseptic or antibiotic treatment was administered during the study.

A total of 49 rats were randomly allocated into seven experimental groups (*n* = 7 per group):

1. NC (Negative Control): No treatment was applied.
2. K: The wound area was treated with standard cream base only.
3. AE: AE extract incorporated into the standard cream base.
4. AW: AW extract incorporated into the standard cream base.
5. AENP: AE extract‐loaded nanoliposomes incorporated into the standard cream base.
6. AWNP: AW extract‐loaded nanoliposomes incorporated into the standard cream base.
7. PC (Positive Control): A commercially available wound‐healing cream (Madecassol) was applied.

The cream base was prepared using glyceryl stearate, liquid paraffin, and propylene glycol in a ratio of 3:1:6 (w/w/w) [33]. AE, AW, AENP, and AWNP were incorporated into the cream base at a concentration of 1% (w/w). All formulations were applied topically once daily in the morning as a thin layer sufficient to completely cover the wound surface. Following application, the wounds were left uncovered throughout the experimental period, and no occlusive dressing or bandage was used. The same application procedure was maintained for all treatment groups to ensure consistency. Wound healing was macroscopically evaluated on days 3, 7, and 14 post‐wounding. Digital photographs of the wounds were taken, and wound areas were quantified using Image J software. The percentage of wound contraction was calculated relative to the initial wound size. Body weights were recorded at the same time points to monitor the general health status of the animals.

For histopathological evaluation, tissue samples were collected from the wound sites [34]. Animals were euthanized by decapitation under deep anesthesia. Excised tissue samples were fixed in 10% neutral buffered formalin and processed using standard histological procedures [30]. Following dehydration through graded alcohol series and clearing with xylene, tissues were embedded in paraffin blocks. Sections of 4–6 μm thickness were obtained using a microtome and mounted on glass slides. The sections were stained with hematoxylin and eosin (H&E) for general histological assessment. Histological evaluation was performed under a light microscope, focusing on key parameters of wound healing, including re‐epithelialization, granulation tissue formation, collagen deposition, inflammatory cell infiltration, angiogenesis, and ulceration. Findings were compared across experimental and control groups.

### 2.7 Animal Ethics Statement

The present study was conducted in compliance with the Animal Welfare Act, the implementing Animal Welfare Regulations, and the principles of the Guide for the Care and Use of Laboratory Animals, National Research Council. The study protocol was approved by the Animal Ethics Committee of Afyon Kocatepe University (number 49533702/56).

### 2.8 Statistical Analysis

To ensure the scientific reliability and reproducibility of the results, all experiments were performed in triplicate or more. Data are presented as mean ± SD. Statistical analyses of the in vitro datasets were performed using GraphPad Prism 9 software, applying one‐way analysis of variance (ANOVA) followed by Tukey's multiple comparison test. Statistical analyses of the in vivo wound‐healing data were performed using SPSS software (version 22.0; IBM Corp., Armonk, NY, USA), applying one‐way ANOVA followed by Duncan's post hoc test. A *p*‐value of <0.05 was considered statistically significant.

## 3 Results

### 3.1 Total Phenolic and Flavonoid Contents of AW and AE Extracts

Total phenolic and flavonoid contents of AW and AE extracts are presented in Table 1. As a result of the tests, the total phenolic content was determined to be 93.15 ± 0.00 mg GAE/g for the AW extract and 157.84 ± 1.16 mg GAE/g for the AE extract. The total flavonoid content was calculated as 6.75 ± 0.18 QE/g for the AW extract and 8.74 ± 0.13 QE/g for the AE extract.

**TABLE 1:** Total phenolic and flavonoid contents of AW and AE extracts.

Extract	**Total phenolic content, mg GAE** a **/g extract**	**Total flavonoid content, mg QE** b **/g extract**
AW	93.15 ± 0.10	6.75 ± 0.18
AE	157.84 ± 1.16	8.74 ± 0.13

*Note*: Values are expressed as mean ± SD of three parallel mea surements (*n* = 3).

a GAE: Gallic acid equivalent.

b QE: Quercetin equivalent.

### 3.2 Antioxidant Activities of AW and AE Extracts

The antioxidant activities of AW and AE extracts are shown in Table 2. The AE extract exhibited higher antioxidant activity than the aqueous extract in all assays. In the DPPH assay, AE demonstrated a lower IC_50_ value than AW, indicating stronger free radical scavenging activity (Table 2). Similarly, CUPRAC, ABTS, and FRAP analyses revealed higher antioxidant capacities for AE. Cupric ion reducing antioxidant capacities of the plant extracts were determined according to the equation (*y* = 0.0397x + 0.3575, R^2^ = 0.9991) as gallic acid equivalent (mg/g extract). The results are given in Table 2. ABTS radical cation scavenging activity of the plant extracts were determined in accordance with the equation (*y* = 0.9198x + 3.5558, R^2^ = 0.9732) of Trolox calibration curve. The results are expressed in terms of Trolox equivalent (mg/g extract) in Table 2. Ferric reducing antioxidant power of the plant extracts were determined according to the equation (*y* = 0.0063x + 0.3599, R^2^ = 0.9830) as Quercetin equivalent (mg/g extract). Also, the results are given in Table 2.

**TABLE 2:** Antioxidant activities of AW and AE extracts.

Parameters	AW	AE	Quercetin
DPPH IC_50_, µg/mL	581.67 ± 0.15	241.82 ± 0.22	16.46 ± 0.10
CUPRAC, mg GAE^a^/g extract	12.36 ± 0.14	16.97 ± 0.16	—
ABTS, mg TE^b^/g extract	81.78 ± 0.25	90.62 ± 0.18	—
FRAP, mg QE^c^/g extract	50.53 ± 0.12	65.73 ± 0.21	—

*Note*: Values are expressed as mean ± SD of three parallel mea surements (*n* = 3).

a GAE: Gallic acid equivalent.

b TE: Trolox equivalent.

c QE: Quercetin equivalent.

### 3.3 In Vitro Cytotoxicity Assay

**FIGURE 1:**
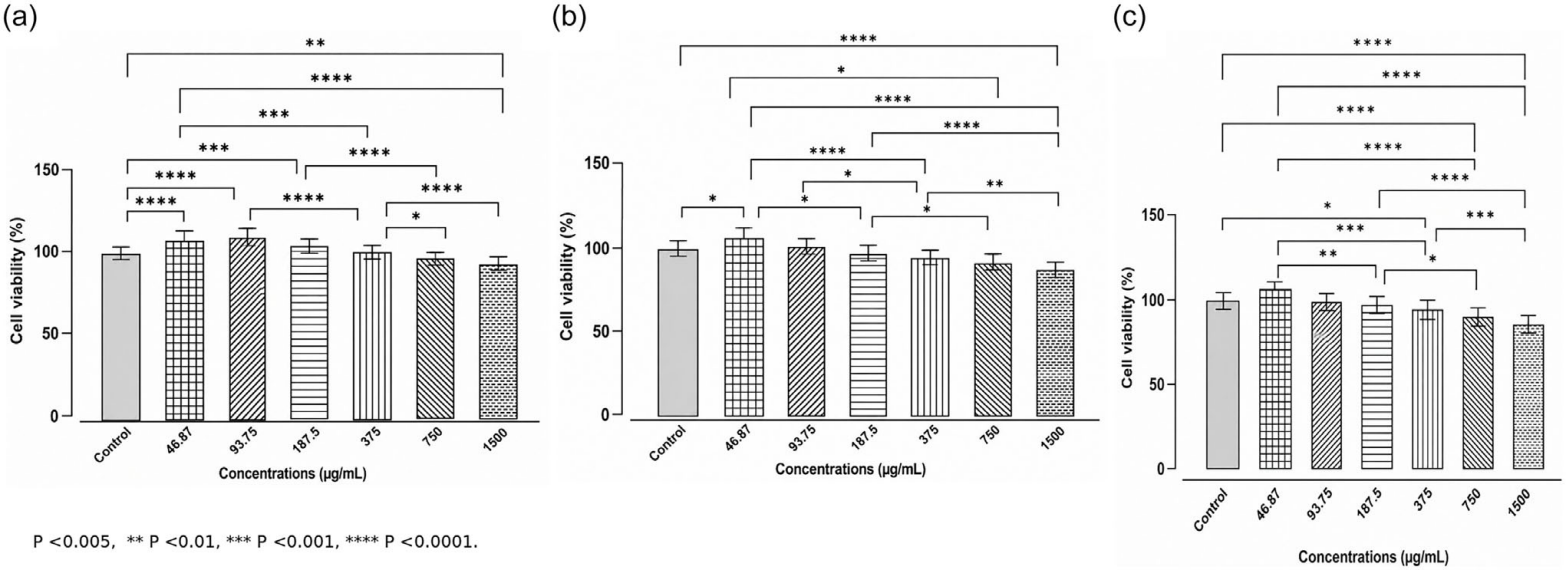
Effect of different concentrations of AE on HDFa cell viability (%) after 24 (a), 48 (b), and 72 h (c) of incubation.

The time‐ and concentration‐dependent effects of AE on HDFa cell viability are shown in Figure 1a,b,c. Following 24, 48, and 72 h of incubation, AE did not induce a significant cytotoxic effect at any of the tested concentrations. Cell viability values remained above the cytotoxic threshold across all time points, although a gradual decrease in viability was observed with prolonged incubation. Statistically significant differences between treatment groups and the control were observed at certain concentrations and time points, as indicated in the figures.

**FIGURE 2:**
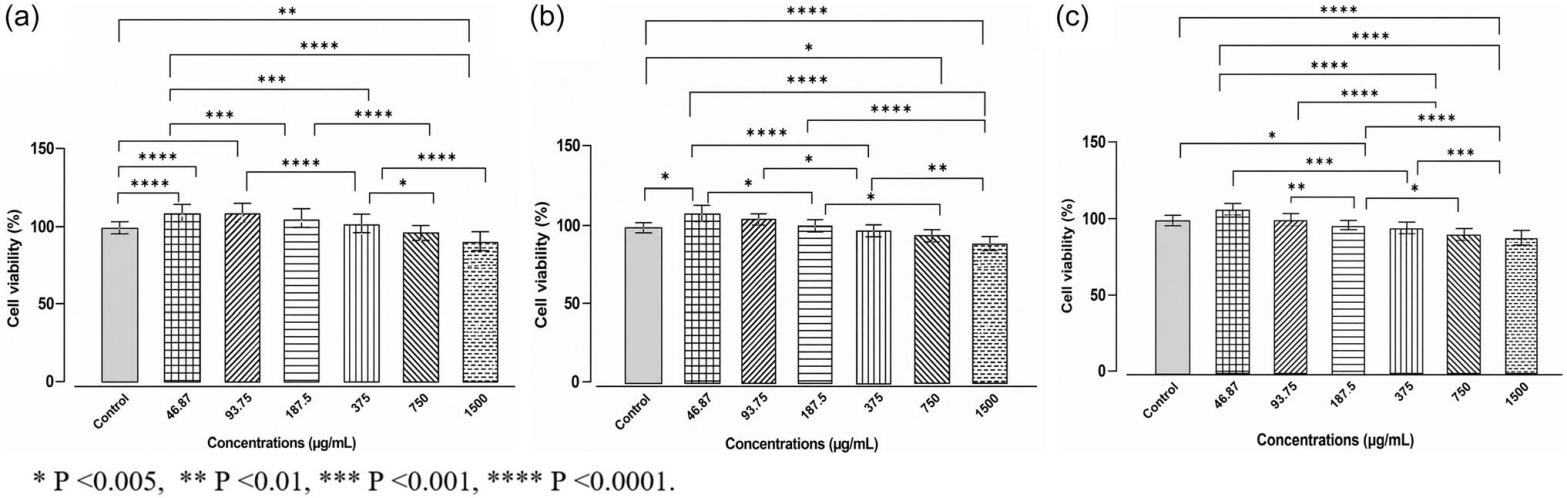
Effect of different concentrations of AW on HDFa cell viability (%) after 24 (a), 48 (b), and 72 h (c) of incubation.

Similarly, the effects of AW on HDFa cell viability at different concentrations and incubation periods are presented in Figure 2a,b,c. AW treatment did not result in cytotoxicity at any concentration tested after 24, 48, or 72 h of exposure. A time‐dependent reduction in cell viability was noted; however, viability percentages remained within the non‐cytotoxic range throughout the experimental period. Comparable trends were observed between AE and AW treated groups.

### 3.4 Nanoliposomes Characterization

**FIGURE 3:**
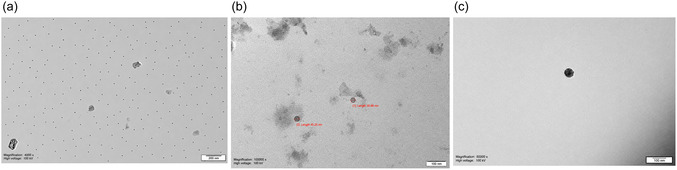
(a,b) SEM and (c) TEM images of AENP.

**FIGURE 4:**
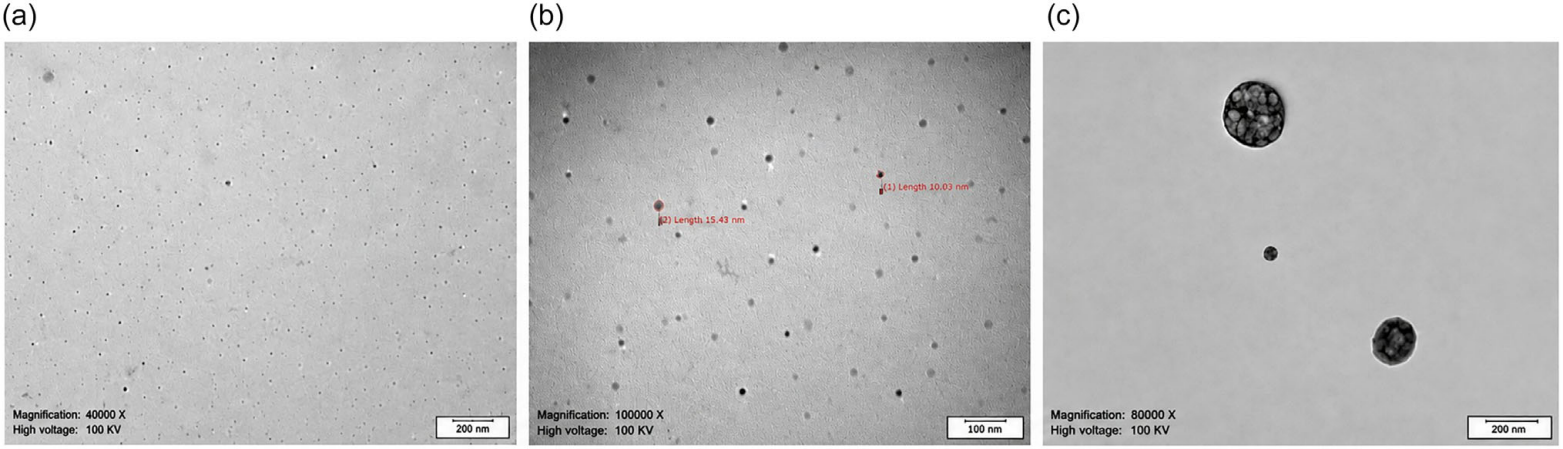
(a,b) SEM and (c) TEM images of AWNP.

SEM and TEM analyses were performed to evaluate the morphological properties of AENP and AWNP. SEM images revealed that both nanoliposome formulations exhibited predominantly spherical morphology with relatively uniform particle distribution and no pronounced structural deformation (Figures 3a,b and 4a,b). The surface of the nanoliposomes appeared compact and continuous, indicating successful nanoliposome formation. TEM micrographs further confirmed the nanoscale size and spherical structure of both AENP and AWNP (Figures 3c, 4c). Compared to SEM observations, TEM images provided clearer visualization of individual nanoliposomes and revealed smaller particle dimensions, reflecting the core size of the nanoliposomes in the dry state. SEM and TEM images confirmed the formation of spherical nanoliposomes in the Nanometer range. Although precise particle size measurements from individual micrographs were not performed, the observed particle dimensions were consistent with the DLS results. The mean hydrodynamic diameters determined by DLS were 231 nm for AENP and 293 nm for AWNP (Figure 7), confirming the nanoscale characteristics of both formulations.

**FIGURE 5:**
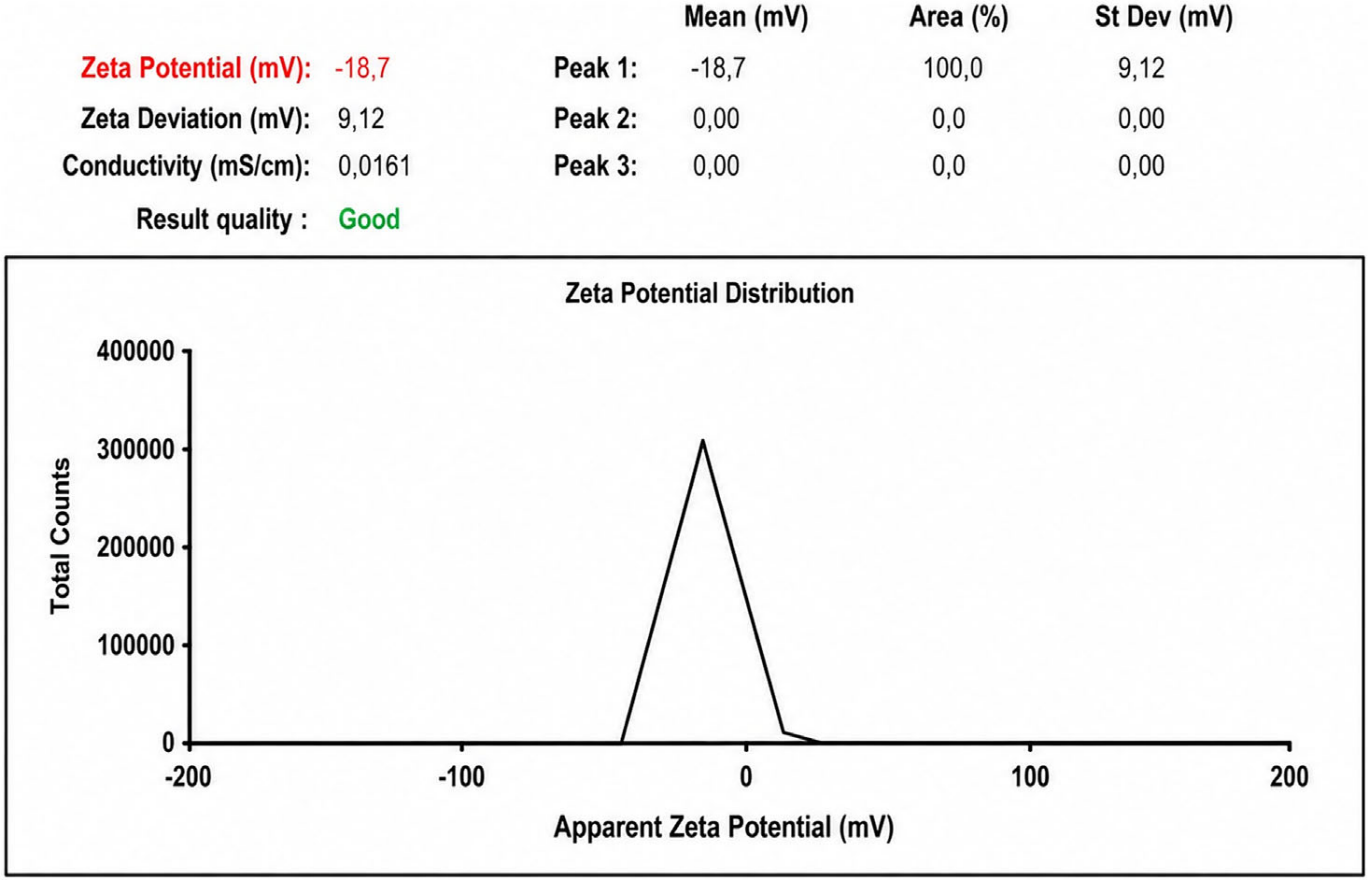
Zeta potential of AENP.

**FIGURE 6:**
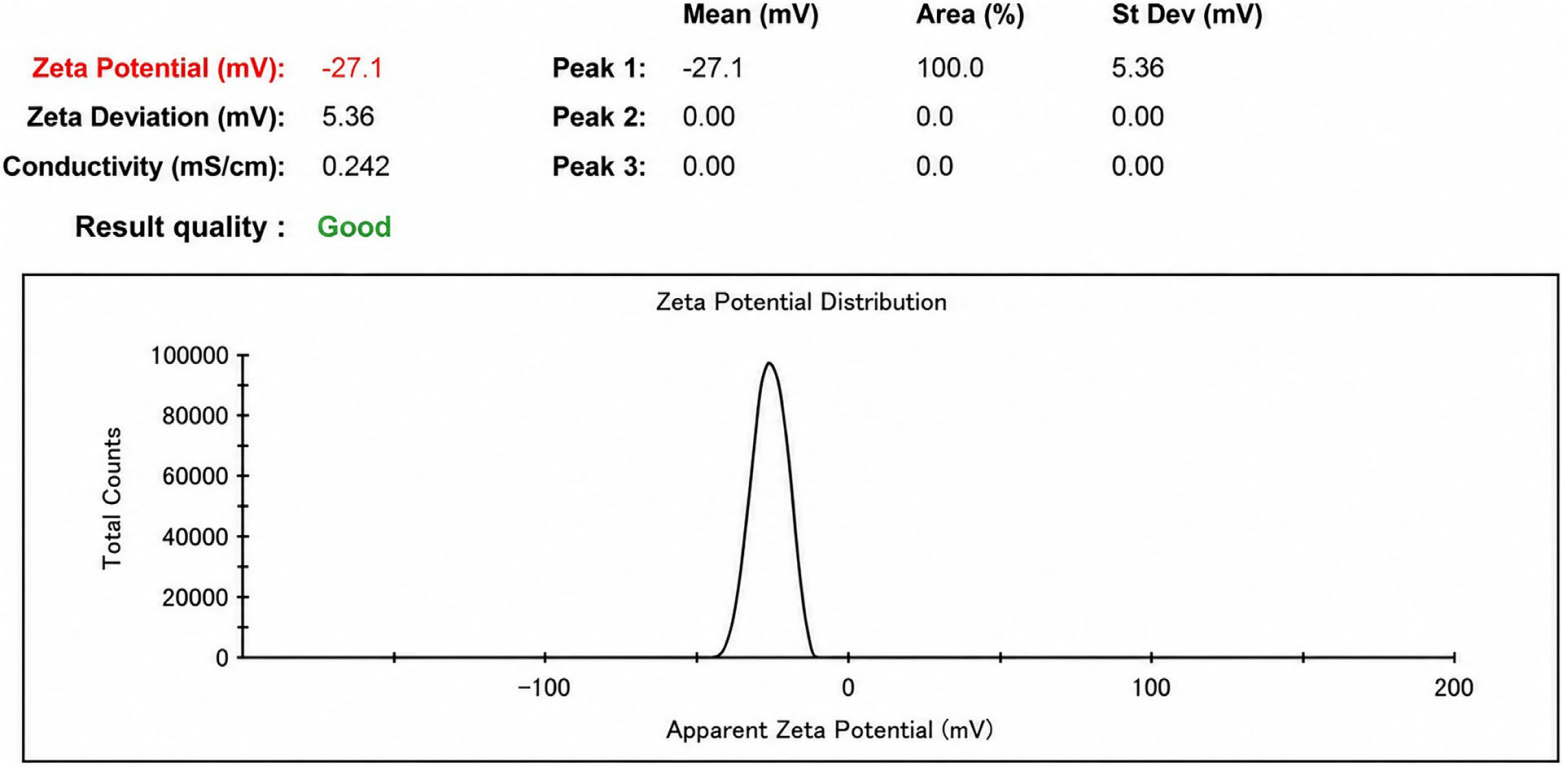
Zeta potential of AWNP.

Particle size and size distribution are among the most critical characteristics of nanoparticulate systems, as they directly influence targeting ability, toxicity, colloidal stability, drug‐loading capacity, and release behavior. In addition, zeta potential is an important parameter for evaluating the stability of colloidal dispersions, with higher absolute values indicating improved electrostatic stabilization. The surface charge properties of the AENP and AWNP were evaluated by zeta potential analysis. The zeta potential of AENP was determined to be −18.7 mV, while AWNP exhibited a zeta potential of −27.1 mV. As shown in Figures 5 and 6, both AENP and AWNP exhibited measurable surface charges, indicating electrostatic stabilization in aqueous suspension. The presence of surface charge suggests that the nanoliposomes possess colloidal stability and reduced tendency toward aggregation.

**FIGURE 7:**
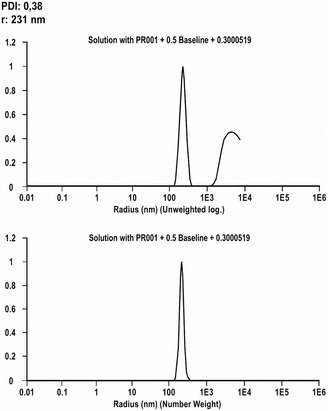
DLS analysis of AENP.

**FIGURE 8:**
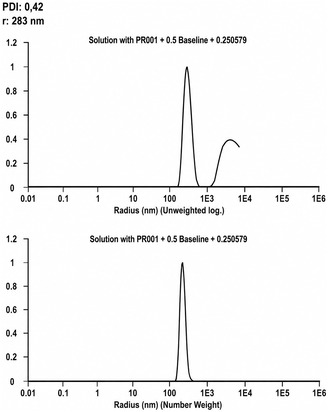
DLS analysis of AWNP.

Dynamic light scattering (DLS) analysis was conducted to determine the hydrodynamic diameter and size distribution of the nanoliposomes in suspension. The PDI values of AENP and AWNP were found to be 0.38 and 0.42, respectively (Figures 7 and 8). The DLS profiles of AENP and AWNP demonstrated a unimodal size distribution, indicating relatively homogeneous nanoliposomes populations. The average hydrodynamic diameters obtained by DLS were larger than those observed in TEM images, which is consistent with the measurement principle of DLS that includes the solvation layer surrounding the nanoliposomes.

### 3.5 In Vitro Nanoliposomes Release Studies

**FIGURE 9:**
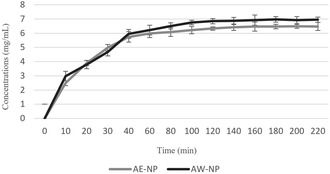
In vitro release profiles of AENP and AWNP.

The in vitro release profiles of AENP and AWNP were determined using calibration curves constructed from the corresponding free extracts. The cumulative release of AENP was calculated based on the calibration equation of the AE extract (*y* = 0.1935x +0,0782, R^2^ = 0.9893), whereas the release of AWNP was quantified using the calibration equation of the AW extract (*y* = 0.2010x + 0.0580, R^2^ = 0.9908). The resulting release profiles are presented in Figure 9. Both formulations exhibited a biphasic release pattern characterized by an initial rapid release phase during the first 40 min, followed by a slower release phase throughout the remainder of the study period. Although the overall release behavior of the two formulations was comparable, AENP showed a higher cumulative release than AWNP at all evaluated time points. These findings suggest that the composition of the encapsulated extract may influence the release behavior of the nanoliposomal formulations.

### 3.6 In Vivo Study Results

**FIGURE 10:**
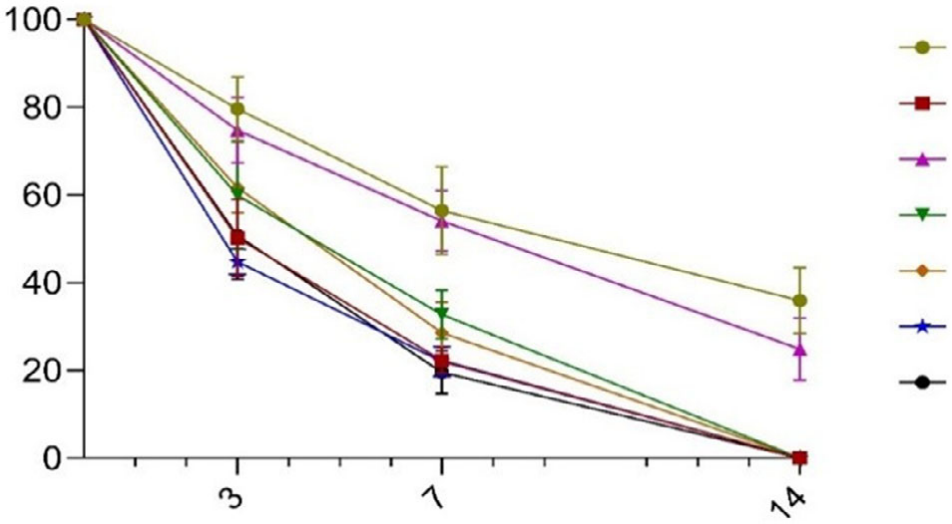
Wound area of application groups.

The in vivo efficacy of the cream base, AE and AW extracts, and the corresponding nanoliposomal formulations (AENP and AWNP) was evaluated using excisional wound model. Digital images of the wound areas were obtained at predetermined time points, and the percentage of wound healing was calculated using Image J software (Figure 10). Based on the ImageJ analysis of the wound healing percentages, the K group exhibited higher healing rates compared to the NC group throughout the treatment period, while showing lower values than the PC group. The AW and AE treated groups demonstrated comparable wound healing percentages. Notably, AENP and AWNP showed higher wound healing rates than their corresponding crude extract groups. The AENP and AWNP groups displayed similar healing profiles; however, the AENP group exhibited the highest overall percentage of wound healing among all experimental groups.

### 3.7 Histopathological Evaluation

**FIGURE 11:**
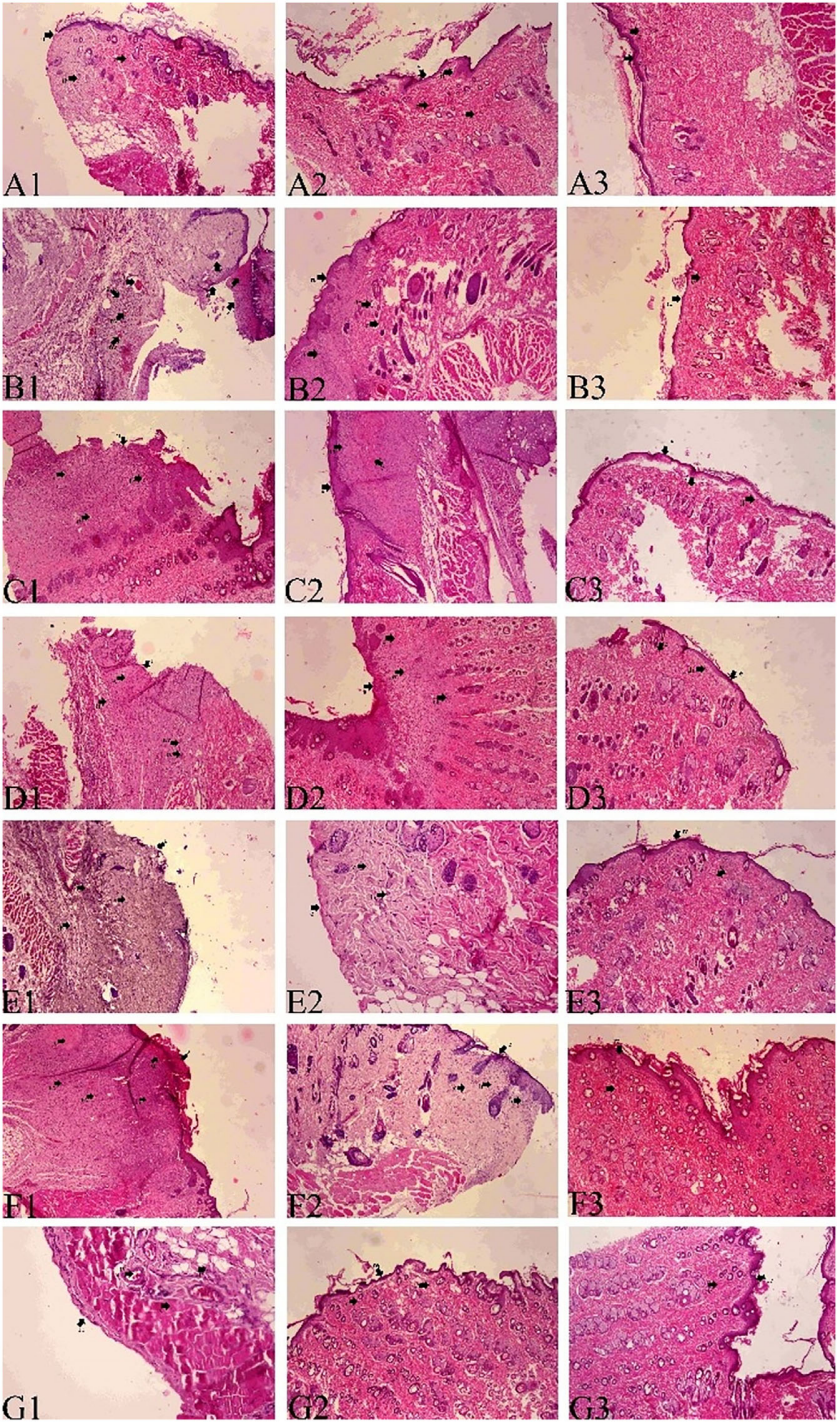
Histopathological evaluation of the in vivo experimental groups at different time points during the wound healing process.

Histopathological analyses were performed on hematoxylin–eosin‐stained tissue sections obtained on days 3, 7, and 14 to assess inflammation, collagen deposition, neovascularization, and re‐epithelialization. The histopathological findings are summarized in Table S1 and illustrated in Figure 11.

#### 3.7.1 Inflammation

Histopathological evaluation revealed significant differences in inflammation severity among the experimental groups at all evaluated time points (*p* < 0.05). Mild inflammation was observed in 80% of the specimens in the AENP and AE groups. In the PC and K groups, mild inflammation was detected in 50% of the samples. In contrast, inflammatory findings were observed in only 10% of the specimens in the NC, AW, and AWNP group, on the 3rd day.

A higher prevalence of mild inflammation was detected in the AENP and AE groups (90%). In the PC and K groups, 80% of the specimens exhibited mild inflammation, while 20% showed moderate inflammation. Inflammatory findings were observed in 60% of the specimens in the NC, AW, and AWNP groups. Specifically, in the NC group, mild inflammation was detected in 50% of the samples on the 7th day.

Severe inflammation persisted in the AE and AENP groups, with 80% of the specimens showing severe inflammation, while 10% exhibited moderate and 10% mild inflammation. In the PC and K groups, severe inflammation was observed in 80% of the samples, with the remaining 20% showing mild inflammation. In the AW and AWNP groups, severe inflammation was detected in 80% of the specimens, whereas 10% exhibited mild inflammation and 10% demonstrated minimal or no inflammatory response. In contrast, the NC group showed predominantly mild inflammation in 50% of the samples, severe inflammation in 30%, and minimal or no inflammation in 20% of the specimens, on the 14th day.

#### 3.7.2 Collagen Deposition

Significant differences in collagen deposition were observed among the experimental groups at all evaluated time points (*p* < 0.05). In the AENP and AE groups, collagen formation was predominantly moderate to high, with 40% of the specimens showing high and 50% moderate collagen density. In comparison, the PC and K groups exhibited higher early collagen deposition, with high collagen density detected in 50% of the specimens. AW and AWNP groups mainly demonstrated moderate collagen deposition, whereas the NC group showed exclusively mild collagen formation on the 3rd day.

A marked increase in collagen deposition was observed in the AENP and AE groups, with high collagen density detected in 80% of the specimens. Similarly, the PC and K groups demonstrated predominantly high collagen levels (90%). In contrast, AW and AWNP groups exhibited mixed moderate and mild collagen deposition, while collagen formation remained consistently mild in the NC group on the 7th day.

During the late healing phase, high collagen deposition persisted in the AENP and AE groups. In contrast, the PC and K groups showed reduced collagen density, with a shift toward moderate levels. AW and AWNP groups predominantly exhibited moderate collagen deposition, whereas the NK group demonstrated mainly mild collagen formation on the 14th day.

#### 3.7.3 Neovascularization

Histopathological assessment revealed significant differences in neovascularization among the experimental groups at all time points (*p* < 0.05). Mild vascular proliferation was predominantly observed in the AENP, AE, and PC groups, whereas AW, AWNP, and K groups also showed mostly mild neovascularization. In contrast, vascular proliferation was detected in only 50% of the NC specimens on the 3rd day.

A pronounced increase in neovascularization was observed in the AENP, AE, and PC groups, with high vascular density detected in 80% of the specimens. AWNP and K groups showed moderate‐to‐high vascular proliferation, whereas the AW group predominantly exhibited low vascular density. The NK group demonstrated minimal neovascularization on the 7th day.

Extensive neovascularization was maintained in the AENP, AE, and PC groups, whereas AW, AWNP, and K groups exhibited sustained but slightly lower vascular density. The NC group demonstrated high vascular proliferation in the majority of specimens, with a minority showing moderate or mild levels, on the 14th day.

#### 3.7.4 Re‐Epithelialization

Re‐epithelialization differed significantly among the experimental groups throughout the healing process (*p* < 0.05). Moderate epithelial regeneration was observed in the AE, AENP, and PC groups, whereas AW and AWNP groups demonstrated mild epithelial formation and the NC group exhibited minimal re‐epithelialization on the 3rd and 7th days.

High re‐epithelialization rates were maintained in the AE and AENP groups, while the PC and K groups demonstrated predominantly high epithelial coverage. AW and AWNP groups exhibited mainly moderate re‐epithelialization, whereas the NC group continued to show incomplete epithelial regeneration on the 14th day.

#### 3.7.5 Scar and Ulcer Formation

Significant differences in scar and ulcer formation were observed among the experimental groups during the early healing phase (*p* < 0.05). High scar and ulcer incidence was detected in the AE and AENP groups, whereas the PC and K groups exhibited predominantly mild alterations. Moderate scar and ulcer formation was observed in the AW, AWNP, and NC groups on the 3rd day.

A reduction in scar and ulcer severity was evident in the AE and AENP groups, with predominantly mild findings observed in the PC and K groups. AW and AWNP groups exhibited mixed mild‐to‐moderate scarring, while the NC group showed persistent moderate scar formation on the 7th day.

By the later stages of healing, scar and ulcer formation was absent in the AE, AENP, and PC groups. Minimal residual scarring was detected in a small proportion of the K and NC groups on the 14th day.

## 4 Discussion

Medicinal and aromatic plants have long been utilized in traditional medicine due to their diverse bioactive constituents, including phenolic compounds, flavonoids, and antioxidants, which are known to modulate multiple biological pathways and exert protective biological effects [35, 36]. In recent years, increasing concerns regarding the adverse effects of synthetic drugs and the global rise in antimicrobial resistance have led to increased scientific attention toward plant‐derived therapeutic agents [37]. In parallel, the search for natural antioxidants has gained importance because of the potential toxic and carcinogenic effects associated with the prolonged use of certain synthetic antioxidants [38]. Numerous studies have reported an association between oxidative stress and impaired tissue repair, highlighting the relevance of identifying natural compounds with antioxidant and regenerative potential [39, 40, 41].

*Anchusa azurea* Miller var. *azurea* is a medicinal plant for which relatively limited information is available regarding its biological activities. Previous studies have reported that *A. azurea* contains appreciable amounts of ascorbic acid, phenolic compounds, and flavonoids, which are closely associated with antioxidant activity [42, 43]. In agreement with these reports, the phytochemical analyses performed in the present study demonstrated that both AW and AE extracts contained considerable amounts of phenolic and flavonoid compounds, with significantly higher levels detected in the AE extract. The phytochemical characterization performed in the present study further supported these observations. The AE extract exhibited markedly higher total phenolic content (157.84 ± 1.16 mg GAE/g extract) and total flavonoid content (8.74 ± 0.13 mg QE/g extract) than the AW extract (93.15 ± 0.10 mg GAE/g extract and 6.75 ± 0.18 mg QE/g extract, respectively). Consistent with these findings, AE demonstrated superior antioxidant activity in all assays performed, including DPPH, ABTS, CUPRAC, and FRAP analyses. Phenolic and flavonoid compounds are well known for their ability to donate hydrogen atoms or electrons and neutralize reactive oxygen species, thereby reducing oxidative stress associated with tissue injury. Previous studies have reported strong correlations between phenolic content and antioxidant activity in medicinal plant extracts, suggesting that these compounds are among the primary contributors to their biological effects [44, 45].

Oxidative stress is recognized as a major factor impairing wound healing by disrupting fibroblast proliferation, extracellular matrix synthesis, angiogenesis, and re‐epithelialization. Therefore, extracts possessing strong antioxidant activity may create a more favorable microenvironment for tissue repair. The enhanced antioxidant capacity observed for AE may partly explain its superior wound healing performance observed in both crude extract and nanoliposomal formulations. Similar relationships between phenolic‐rich plant extracts, antioxidant activity, and accelerated wound closure have been reported in several experimental wound healing studies [9, 46, 47].

The higher antioxidant activity observed in the AE extract is likely associated with its greater total phenolic and flavonoid contents compared with the AW extract. Phenolic and flavonoid compounds are recognized as major contributors to antioxidant activity due to their ability to donate electrons or hydrogen atoms and neutralize reactive oxygen species [38]. Consistent with these findings, the AE extract exhibited superior antioxidant activity in the DPPH, ABTS, CUPRAC, and FRAP assays compared with the AW extract. The higher antioxidant capacity of the AE extract may be attributed to its greater phenolic and flavonoid contents, which are known to play important roles in scavenging reactive oxygen species. Since excessive oxidative stress can impair collagen synthesis, cell migration, and re‐epithelialization during wound healing, the stronger antioxidant activity of the AE extract may have contributed to the enhanced wound‐healing effects observed in the present study [10, 13].

In this study, the cytotoxicity profile of AE on HDFa cells was evaluated in a time‐ and concentration‐dependent manner over 24, 48, and 72 h. The results indicate that AE did not induce significant cytotoxic effects at any tested concentration, with cell viability remaining above the commonly accepted cytotoxic threshold at all time points. Although a gradual decline in viability was observed with prolonged incubation, certain concentrations and time points showed statistically significant differences compared to the control, suggesting minor, biologically tolerable effects. These findings indicate that AE is generally biocompatible with human dermal fibroblasts at the tested doses, supporting its potential safety for topical or biomedical applications. In another study, the cytotoxicity of polyphenolic‐rich extracts of *Anchusa officinalis* on mouse fibroblast (NCTC L929) cells was examined; the extract did not show significant cytotoxic effects at concentrations up to 250 µg/mL, although higher concentrations induced reduced viability [48]. This supports the notion that the observed slight decrease in HDFa viability likely reflects physiological adaptation rather than cytotoxic injury. Overall, both our experimental results and the literature indicate that AE preserves the viability of dermal fibroblasts, a profile that is favorable for wound healing applications where fibroblast functionality is critical.

AE‐ and AW‐loaded nanoliposomes were obtained, and the combined SEM, TEM, zeta potential, and DLS analyses provided comprehensive insight into the physicochemical characteristics of AENP and AWNP. SEM and TEM imaging confirmed that both nanoliposomes systems were successfully fabricated with predominantly spherical morphology and nanoscale dimensions, as morphological analysis by electron microscopy is a standard and reliable approach for assessing nanoliposome shape and size. The absence of severe aggregation or irregular morphology suggests that the formulation process was effective and reproducible, consistent with previous reports on plant extract–based nanoliposome syntheses where controlled morphology was achieved [49, 50].

Zeta potential analysis revealed that both nanoliposomal formulations possessed negative surface charges, indicating electrostatic stabilization in aqueous suspension. The zeta potential value of AENP (−18.7 mV) suggests moderate colloidal stability, whereas the more negative value observed for AWNP (−27.1 mV) indicates comparatively stronger electrostatic repulsion and improved dispersion stability. It has been widely reported that lipid‐based nanosystems exhibiting zeta potential values in this range can maintain sufficient colloidal stability for topical and biomedical applications, particularly in plant extract–loaded formulations [20, 51].

DLS results demonstrated that AENP possessed a smaller hydrodynamic radius (231 nm) compared to AWNP (283 nm), suggesting that the AE‐loaded formulation forms more compact nanoliposome structures in aqueous media. Polydispersity index (PDI) values of 0.38 for AENP and 0.42 for AWNP indicate moderate polydispersity, which is frequently reported in plant extract–loaded nanoliposome systems. The slightly higher PDI for AWNP suggests a broader size distribution, which may be attributed to differences in extract composition and its interaction with the nanoliposome matrix.

Overall, the physicochemical properties observed for AENP and AWNP suggest that these nanoliposome systems are structurally stable and suitable for further biological evaluation, aligning with trends reported for extract‐based nanoscale formulations in recent literature.

The in vivo wound healing experiments demonstrated that both crude extracts and nanoliposomal formulations enhanced wound closure compared to the negative control group. Notably, the nanoliposomal formulations demonstrated enhanced wound healing outcomes relative to their corresponding crude extracts, as supported by macroscopic wound measurements, ImageJ analysis, and histopathological evaluation. This improvement may be associated with the controlled and sustained release of bioactive constituents from the nanoliposomal carriers, resulting in prolonged exposure of the wound tissue to therapeutically relevant concentrations. Nano‐drug delivery systems, including lipid‐based carriers such as liposomes and nanoliposomes, have been reported to protect bioactive agents from degradation, improve stability, and enable sustained release during wound healing, ultimately enhancing therapeutic efficacy relative to conventional formulations [52, 53].

Wound healing is a highly coordinated process involving overlapping inflammatory, proliferative, and remodeling phases. Histopathological parameters such as inflammation, collagen deposition, neovascularization, and re‐epithelialization are widely accepted indicators for evaluating the quality and progression of tissue repair [54, 55]. In the present study, observable differences among experimental groups were noted at all evaluated time points, suggesting that the applied treatments modulated the wound healing response.

Inflammation represents an essential and tightly regulated phase of wound healing, facilitating debris clearance and activation of molecular signaling pathways required for subsequent tissue regeneration. In the present study, the AE and AENP groups exhibited a more pronounced inflammatory response, particularly at later time points. Rather than indicating impaired healing, this sustained inflammatory activity may reflect a controlled and pro‐regenerative immune response that supports angiogenesis, fibroblast activation, and extracellular matrix remodeling. Previous studies have demonstrated that appropriately regulated inflammation is critical for effective wound repair and that insufficient or delayed inflammatory signaling may impair tissue regeneration [46]. Importantly, in the present study, the enhanced inflammatory response observed in the AE and AENP groups was accompanied by improved collagen deposition, neovascularization, and re‐epithelialization, indicating coordinated progression of the healing process.

Collagen deposition is a key feature of the proliferative phase and plays a critical role in restoring tissue integrity and mechanical strength. The AE and AENP groups demonstrated sustained collagen deposition from day 7 through day 14, suggesting active fibroblast function and extracellular matrix synthesis. Increased collagen deposition and improved epithelialization have been reported with nanoliposome‐loaded phytochemical systems in vivo, supporting the role of nanocarrier systems in promoting these aspects of healing [53]. Although excessive collagen accumulation has been associated with hypertrophic scarring, the concurrent reduction in scar formation observed at later stages suggests that collagen remodeling progressed in a regulated manner. The PC and K groups exhibited higher collagen deposition at early time points followed by a transition toward moderate levels, consistent with progression into the remodeling phase. In contrast, the NC group consistently demonstrated mild collagen deposition, which is consistent with previous reports describing delayed fibroplasia in untreated wounds.

Angiogenesis plays a central role in supplying oxygen and nutrients to regenerating tissue and is closely linked to collagen synthesis and epithelial regeneration. In this study, increased neovascularization was observed in the AE, AENP, and PC groups from day 7 onward, with sustained vascular presence through day 14. These findings suggest an active proliferative phase and are consistent with previous studies reporting that enhanced angiogenesis is associated with accelerated wound healing [55, 56, 57, 58]. The delayed and less pronounced angiogenic response observed in the NC group further supports the conclusion that untreated wounds undergo slower tissue regeneration.

Re‐epithelialization is critical for restoring the epidermal barrier and protecting the wound from external insults. The AE and AENP groups exhibited advanced re‐epithelialization, with high epithelial coverage maintained at later stages. This observation suggests effective keratinocyte migration and proliferation, potentially supported by enhanced angiogenesis and extracellular matrix formation. In contrast, the NC group showed delayed and incomplete re‐epithelialization, consistent with reduced regenerative signaling.

Scar formation is an inevitable outcome of adult wound healing; however, its extent depends on the balance between inflammation, collagen deposition, and tissue remodeling. In the present study, early scar and ulcer formation were more evident in the AE and AENP groups, which may reflect active early tissue remodeling. Importantly, scar formation decreased at later stages, suggesting progression toward effective remodeling and tissue organization. Persistent mild scarring observed in the NC and K groups may be associated with delayed resolution of inflammation and incomplete matrix remodeling.

Overall, the histopathological findings indicate that AE and AENP treatments influenced multiple stages of the wound healing process in a coordinated manner. Although an enhanced inflammatory response was observed, it was accompanied by increased angiogenesis, collagen deposition, and re‐epithelialization, ultimately supporting effective tissue regeneration.

The present study has several limitations that should be acknowledged. Encapsulation efficiency, loading capacity, and storage stability of the nanoliposomal formulations were not determined and therefore could not be correlated with the observed biological activities. In addition, although SEM and TEM analyses confirmed the formation of spherical nanoscale vesicular structures, direct confirmation of the phospholipid bilayer architecture was not achieved. Advanced characterization techniques such as high‐resolution TEM or cryo‐TEM would be required to verify the bilayer structure of the nanoliposomes. Furthermore, the in vitro release evaluation was performed using direct dispersion in PBS rather than a dialysis‐based separation system and was limited to a relatively short experimental period (300 min). Therefore, the obtained release profiles should be interpreted as comparative release behavior of the formulations rather than definitive diffusion‐controlled release profiles. Future studies should include comprehensive physicochemical characterization, determination of encapsulation parameters, long‐term stability assessment, bilayer structure verification, and extended‐release investigations using more rigorous release methodologies to further validate the therapeutic potential of these formulations.

## 5 Conclusion

In conclusion, the present study demonstrated that aqueous and hydroethanolic extracts of *Anchusa azurea* Miller var. *azurea* and their corresponding nanoliposomal formulations exhibit promising wound‐healing potential. Phytochemical characterization revealed that the hydroethanolic extract possessed higher phenolic and flavonoid contents than the aqueous extract, which was accompanied by stronger antioxidant activity in DPPH, ABTS, CUPRAC, and FRAP assays. These findings suggest that the biological effects of the extracts may be associated with their rich phenolic composition and antioxidant properties.

The successful formulation and characterization of extract‐loaded nanoliposomes resulted in nanoscale delivery systems with suitable physicochemical properties for topical application. Both crude extracts and nanoliposomal formulations significantly improved wound healing in the in vivo excisional wound model compared with the negative control group. Notably, nanoliposomal formulations demonstrated greater wound‐healing efficacy than their corresponding crude extracts, indicating that nanoliposomal delivery may enhance the therapeutic performance of *A. azurea* extracts.

Among the tested formulations, the AENP‐loaded wound cream exhibited the highest wound‐healing activity, while both AENP and AWNP produced healing outcomes comparable to the positive control. Histopathological findings further confirmed enhanced collagen deposition, neovascularization, and re‐epithelialization in the treatment groups. Collectively, these results indicate that *A. azurea*, particularly in nanoliposomal form, represents a promising natural source for the development of wound‐care formulations. Further studies focusing on the identification of active constituents, molecular mechanisms underlying wound repair, and long‐term efficacy and safety evaluations are warranted to support its potential therapeutic application.

## Author Contributions

**Sevim Feyza E** **rdoğmuş** **:** investigation, writing – review and editing, methodology, software, formal analysis, visualization. **Hasan Hüseyin** **Demirel**: conceptualization, investigation, writing – original draft, methodology, formal analysis. **Özlem Erdal Altıntaş**: investigation, methodology, validation. **İffet İrem Tatlı Çankaya**: investigation, methodology, validation. **Nurullah O** **kumuş**: investigation, methodology.

## Funding

The study was supported by the Türkiye Bilimsel ve Teknolojik Araştırma Kurumu.

## Conflicts of Interest

The authors declare no conflicts of interest.

## Supporting information

Supplementary Material

## Data Availability

The data that support the findings of this study are available on request from the corresponding author. The data are not publicly available due to privacy or ethical restrictions.
