# Supplementary material for: Exploring the Wound‐Healing Properties of Anchusa azurea: A Nanoliposome‐Based Approach
Source: ChemistryOpen. 2026 Aug 13;15(8):e70254. doi: 10.1002/open.70254 (PMC13473703; doi:10.1002/open.70254)
Supplement: Supplementary file 1 [file OPEN-15-e70254-s001.pdf]

Groups-Treathment Days	Re-epithalization	Neovascularisation	Scar and Ulcus	Collagen	Inflamatuar cell
K-3.day	0,00±0,00^{j}	0,16±0,41^{h}	2,01±0,24^{b}	0,51±0,22^{ı}	1,51±0,54^{bcd}
K-7.day	0,33±0,51^{ıj}	0,83±0,41^{g}	1,33±0,51^{c}	0,83±0,28^{ghı}	1,51±0,54^{bcd}
K-14.day	1,66±0,51^{cde}	1,33±0,51^{defg}	0,00±0,00^{f}	1,51±0,54^{cdef}	0,66±0,51^{fgh}
NC-3.day	0,00±0,00^{j}	0,16±0,41^{h}	2,51±0,54^{a}	0,51±*,21^{ı}	2,83±0,41^{a}
NC-7.day	0,83±0,41 ^{ghı}	1,01±0,02^{fg}	2,01±0,23^{b}	0,66±0,28^{hı}	1,83±0,41^{be}
NC-14.day	1,51±0,54 ^{cdef}	1,51±0,54^{cdef}	0,00±0,00^{f}	1,01±0,12^{fghı}	0,83±0,38^{efg}
AW-3.day	0,00±0,00 ^{j}	1,01±0,20^{fg}	1,33±0,51^{c}	1,16±0,41^{efgh}	2,01±0,63^{b}
AW-7.day	1,01±0,01 ^{fgh}	1,33±0,51^{defg}	0,83±0,42^{cde}	1,66±0,51^{cde}	2,01±0,63^{b}
AW-14.day	1,51±0,54 ^{cdef}	1,51±0,54^{cdef}	0,00±0,00^{f}	1,33±0,52^{defg}	0,66±0,51^{fgh}
AE-3.day	1,83±0,40 ^{cd}	1,16±0,41^{efg}	1,33±0,51^{c}	1,01±0,12^{fghı}	1,51±0,83^{bcd}
AE-7.day	1,33±0,51 ^{defg}	1,51±0,54^{cdef}	0,83±0,32^{ef}	1,33±0,51^{defg}	1,01±0,12^{def}
AE-14.day	1,66±0,51 ^{cde}	1,16±0,51^{bcde}	0,00±0,00^{f}	1,51±0,54^{cdef}	0,33±0,51^{gh}
AWNP-3.day	0,51±0,54 ^{hıj}	1,01±0,06^{fg}	1,33±0,51^{cd}	1,33±0,51^{defg}	1,33±0,54^{cde}
AWNP-7.day	1,83±0,41 ^{cd}	1,33±0,51^{defg}	0,51±0,32^{cde}	1,51±0,54^{cdef}	1,01±0,12^{def}
AWNP-14.day	2,51±0,83 ^{ab}	2,33±0,51^{a}	0,00±0,00^{f}	2,01±0,62^{bc}	0,83±0,41^{efg}
AENP-3.day	0,51±0,54 ^{hıj}	1,51±0,54^{cdef}	0,83±0,41^{cde}	1,33±0,51^{defg}	1,16±0,41^{def}
AENP-7.day	1,16±0,41 ^{efg}	1,84±0,41^{abcd}	0,51±0,16^{ef}	1,83±0,41^{bcd}	0,66±0,51^{fgh}
AENP-14.day	2,66±0,51 ^{a}	2,16±0,41^{ab}	0,00±0,00^{f}	2,33±0,51^{b}	0,66±0,51^{fgh}
PC-3.day	0,33±0,05 ^{ıj}	1,33±0,51^{defg}	0,66±0,12^{de}	1,16±0,41^{efgh}	1,16±0,39^{def}
PC-7.day	2,01±0,05 ^{bc}	2,01±0,08^{abc}	0,51±0,32^{ef}	1,83±0,42^{bcd}	1,16±0,41^{def}
PC-14.day	3,01±0,06 ^{a}	1,83±0,04^{bcd}	0,00±0,00^{f}	3,01±0,52^{a}	1,01±0,12^{h}
P değeri	$p < 0,05$	$p < 0,05$	$p < 0,05$	$p < 0,05$	$p < 0,05$

Table S1. Comparison of the mean histological scores of the wounds in the treatment and control groups.
